# Regulating Effect of Cement Accelerator on High Content Solid-Wastes Autoclaved Aerated Concrete (HCS-AAC) Slurry Performance and Subsequent Influence

**DOI:** 10.3390/ma14040799

**Published:** 2021-02-08

**Authors:** Dingkun Xie, Lixiong Cai, Jie Wang

**Affiliations:** 1 School of Civil Engineering and Architecture, Wuhan University of Technology, Wuhan 430070, China; xiedingkun@whut.edu.cn; 2 School of Civil and Hydraulic Engineering, Huazhong University of Science and Technology, Wuhan 430074, China; 3 State Key Laboratory of Silicate Materials for Architectures, Wuhan University of Technology, Wuhan 430070, China; wangjie_bme@whut.edu.cn

**Keywords:** carbide slag, autoclaved aerated concrete, cement accelerator, foaming and thickening process, physical-mechanical properties, hydration products

## Abstract

Adverse side-effects occurred in slurry foaming and thickening process when carbide slag was substituted for quicklime in HCS-AAC. Cement accelerators were introduced to modify the slurry foaming and coagulating process during pre-curing. Meanwhile, the affiliated effects on the physical-mechanical properties and hydration products were discussed to evaluate the applicability and influence of the cement accelerator. The hydration products were characterized by mineralogical (XRD) and thermal analysis (DSC-TG). The results indicated that substituting carbide slag for quicklime retarded slurry foaming and curing progress; meanwhile, the induced mechanical property declination had a negative effect on the generation of C–S–H (I) and tobermorite. Na_2_SO_4_ and Na_2_O·2.0SiO_2_ can effectively accelerate the slurry foaming rate, but the promoting effect on slurry thickening was inconspicuous. The compressive strength of HCS-AAC obviously declined with increasing cement coagulant content, which was mainly ascribed to the decrease in bulk density caused by the accelerating effect on the slurry foaming process. Dosing Na_2_SO_4_ under 0.4% has little effect on the generation of strength contributing to hydration products while the addition of Na_2_O·2.0SiO_2_ can accelerate the generation and crystallization of C–S–H, which contributed to the high activity gelatinous SiO_2_ generated from the reaction between Na_2_O·2.0SiO_2_ and Ca(OH)_2_.

## 1. Introduction

Autoclaved aerated concrete (AAC) is a commonly used wall material with good thermal insulation, which is attributed to its fine, uniform, independent pore structure [1,2,3]. Conventional commercial AAC production usually uses natural silica sand or high pozzolanic active material; fly ash is used as siliceous material to provide Si or Al in hydrothermal synthesis reactions [4,5]. Meanwhile, portland cement and aluminum (Al) paste were used as cementitious material and a gas-generating agent, respectively [5]. Gypsum was treated as autoclaving auxiliary agent to accelerate the formation of target hydration products during the autoclave process [6]. The most commonly used calcareous material in AAC was quicklime, which not only supplies calcium ions for hydrothermal synthesis reaction in the autoclaving process, but also provides uniform heat, which directly affects the rough-body stability and pre-curing process duration [4,5].

Energy consumption and environmental protection are two major issues of great concern to the building materials industry. In order to improve the sustainability of AAC production, research into industrial solid waste substitutes covered almost all of the abovementioned raw materials. Cement was used to generate the initial rough-body strength, which can be replaced by alkali-activated volcanic material [7]. As a result of the activation effect of autoclaving, the substitution of siliceous solid wastes for traditional siliceous materials, such as blast-furnace slag [8], coal bottom ash [9], copper tailing [10], coal gangue [11] and iron tailing [12] has been investigated. Because the main ingredient of phosphogypsum (PG) is calcium sulphate dehydrate (CaSO_4_·H_2_O), it can be used as autoclaving auxiliary agent. The calcination preparation process determined quicklime to be the most energy-consuming raw material in AAC preparation [13] and that it can be substituted by carbide slag, the major byproduct of acetylene (C_2_H_2_) production, which is over 85% calcium hydroxide (Ca(OH)_2_) [14]. Therefore, high-content solid-wastes autoclaved aerated concrete (HCS-AAC) has been proposed not only to transform “waste” into “wealth”, but also to reduce production costs [6]. The complete substitution of siliceous material and auxiliary material by industrial solid wastes will not affect the formation process of the AAC rough body significantly. However, complete substitution of carbide slag for quick lime, which resulted in the absence of uniform quicklime hydration heat, had a severe effect on the formation of rough-body porosity, and this not only slowed down the reaction rate of Al paste and the slurry foaming rate, but also reduced the thickening speed of the rough body [15].

Most of the previous studies on carbide slag-based AAC were concentrated on final product performance. Yuli Wang et al. utilized 30–40% high-volume desulfurization fly ash and 8% carbide slag to replace natural gypsum and quicklime, which produced 600 kg/m^3^-grade AAC with 3.5 MPa in compressive strength [16]. Fan Junjie et al. [17] completely replaced quicklime with carbide slag and prepared AAC products over 600 kg/m^3^ with only 2.0 MPa, which cannot satisfy the requirement of B06 A3.5-grade product (bulk density ≤625 kg/m^3^, compressive strength ≥3.5 MPa) stipulated in GB 11968-2006, the Chinese national standard. Changlong Wang et al. [12] prepared 600 kg/m^3^, 4.4 MPa-grade AAC products with 60% high silicon iron tailing as the main siliceous material, 25% carbide slag as the calcareous material and discussed the effect of the substituted carbide slag ratio, fineness and pre-curing temperature on the physical-mechanical properties. But the effect of carbide slag substitution on the foaming and thickening processes that determine the formation stability and quality of porous structures were usually neglected, to say nothing of the regulation of the pre-curing process.

In response to the unmatched slurry foaming and thickening process and the overlong pre-curing problem caused by substituting carbide slag for quicklime, the authors introduce fast hardening sulphate aluminum cement (SAC) into AAC production and achieved positive results in foaming and thickening adjustment [15]. Otherwise, the microwave heating method was used to compensate for the absence of a uniform heat source in carbide slag-based AAC. The slurry foaming rate nearly doubled while the pre-curing duration was shortened by 0.5–1 h [18]. The gas generation of AAC is mainly affected by slurry temperature and alkalinity while rough-body thickening is mainly affected by the hydration rate of the cementitious materials. Adding a cement accelerator to expedite early-age cement hydration may be another worthwhile approach for modifying the foaming and thickening problem of the AAC rough body. In concrete, industrial, mineral and chemical accelerators have been commonly utilized in a variety of engineering fields to regulate the work performance [19] such as spray concrete [20,21,22], cellular concrete [23], urgent repair concrete [24]. However, the explicit effect of a cement accelerator on an HCS-AAC slurry’s performance, physical-mechanical properties and hydration products is still unclear and needs to be clarified.

This study introduced the frequently used cement accelerator Na_2_SO_4_ and Na_2_O·2.0SiO_2_ in HCS-AAC as a pre-curing process adjustment agent and mainly discussed the influence on the slurry’s foaming property and time-dependent rheological behavior to evaluate the feasibility of adjusting the foaming and thickening process. Meanwhile, the effects on the physical-mechanical properties were determined by detecting bulk density and compressive strength to represent the side effect of service performance. Additionally, analyses of the mineralogical (XRD) and thermal characteristics (DSC-TG) were conducted to show the microscopic effects of cement accelerators on HCS-AAC. Finally, the influence of various cement coagulants on slurry performance, physical-mechanical properties and hydration products was discussed and analyzed. The results of this paper can provide theoretical and technological guidance for the slurry foaming and rough-body thickening process modification of carbide slag HCS-AAC.

## 2. Materials and Methods

### 2.1. Raw Materials

The raw materials utilized in this study include 42.5 Portland cement (PO 42.5), quicklime, carbide slag, iron tailing, quartz tailing and PG: chemical compositions are presented in Table 1 and granularity characteristics are shown in Table 2. The PO 42.5 (Wuhan, Hubei, China) was used as a binding material, which is indispensable for generating rough-body initial strength for the mold stripping and cutting operation. PO 42.5 came from the Yadong Cement Co., Ltd. in Wuhan. It has a 210 min initial set and 289 min final set. Quicklime (Huangshi, Hubei, China) and carbide slag (Yichang, Hubei, China) were the calcareous materials that supplied calcium ions for the hydrothermal synthesis reaction with siliceous material. The quicklime had 71.6% active calcium oxide (CaO) and a 9.3% residue weight on a 80 μm screen sieve; meanwhile, the digestion time and temperature were 12 min and 87 °C, respectively. The carbide slag was obtained from Yichang Chemical Industry Co., Ltd. and contained 35–50% moisture in its original state and 87% Ca(OH)_2_ after desiccation. Siliceous materials, iron tailing and quartz tailing were the accessory products of ore dressing and quartz processing that were obtained from Huangshi and Suizhou, China, respectively. The iron tailing, which only contained 42.9% SiO_2,_ was regarded as a low silicon tailing for AAC production. The quartz tailing had 93% crystal quartz which made it an excellent silicon supplement material to make up for the silicon deficiency of the iron tailing. Regarding other materials, the PG was from the phosphorus fertilizer plant Chunxiang Chemical Industry Co., Ltd. (Jingmen, Hubei, China). The foaming agent was Al paste (Zibo, Shandong, China) which has 80% solid content, 86% active Al content, and a 5417 cm^2^/g coating surface on water. The coagulants included anhydrous sodium sulfate (Na_2_SO_4_) and sodium silicate solution (Na_2_O·2.0SiO_2_) which were usually used as cement accelerator. Na_2_SO_4_ and Na_2_O·2.0SiO_2_ were produced by Shanghai Sinopharm Chemical Reagent Co., Ltd. with 99% purity and 50% solid content, respectively.

### 2.2. Mixture Design and Preparation Procedure

#### 2.2.1. Mixture Design

According to a previous study by the authors on carbide slag AAC [6,12], the basic control group was PO 42.5 (10%), calcareous material (31%), siliceous mix powder (59%), Al paste (0.14%) and a water-to-powder ratio (W/P) (0.54). As shown in Table 3, the QL-AAC and CS-AAC mixtures were conducted as a comparison group and control group, respectively, to demonstrate the slurry performance, physical-mechanical properties and hydration product characteristics of quicklime and carbide slag AAC. Different dosages of the commonly used cement accelerators, Na_2_SO_4_ (0.1%, 0.2%, 0.3%, 0.4%) and Na_2_O·2.0SiO_2_ (0.25%, 0.5%, 1%, 1.5%), in relative proportion to PO 42.5 into basic control group so that the multidimensional effect on carbide slag HCS-AAC slurry and products could be investigated.

#### 2.2.2. Preparation Procedure

**Figure 1:**
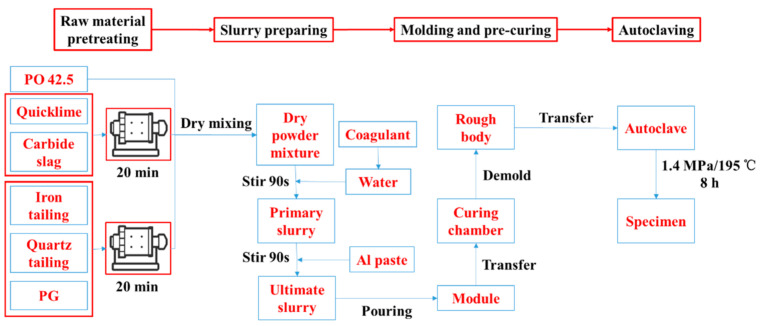
Flow chart of specimen preparation procedure.

The HCS-AAC was prepared by following the procedure presented in Figure 1, which includes 4 stages: raw material pretreating, slurry preparing, molding and pre-curing and autoclaving. The specific process was presented as follows:

(1) Raw material pretreating

Calcareous materials were ground separately in a φ500 mm × 500 mm cylinder experimental ball mill for 20 min. Siliceous materials and PG were mixed according to the mass ratio in Table 3 (28:28:3) before being ground in same ball mill for 20 min [6]. Afterward, PO 42.5, calcareous material and siliceous materials were mixed in a V-type blender to create a dry powder mixture for slurry preparation.

(2) Slurry preparation

The primary slurry was obtained by combining the dry powder mixture proportionately with 50 ± 1 °C warm water dissolved with an accelerator and stirred for 90 s. Then, Al paste was added and it was stirred for another 90 s to generate the ultimate slurry for molding.

(3) Molding and pre-curing

After that, the ultimate slurry was poured into molds that were kept at 55 ± 1 °C and 70% relative humidity (RH) in an environmental control chamber for foaming and pre-curing until the rough body was strong enough to go through demolding and transferring.

(4) Autoclaving

Finally, the rough body was transferred into an autoclave and charged with steam to achieve a saturated vapor pressure environment. The curing temperature and pressure in the autoclave was 195 °C and 1.4 MPa, respectively. It was held in the curing environment for 8 h and cooled to room temperature before the final specimens were obtained.

### 2.3. Characterization Methods

#### 2.3.1. Raw Material Characteristics

The granular characteristics of powdery raw materials were measured according to the methods stipulated in GB/T 8074-2008 and GB/T 14684-2011 for specific surface area and screen residue weight, respectively. The oxides composition was detected by a Zetium X-ray fluorescence spectrometer (XRF) (PANalytical.B.V, Netherlands); meanwhile, the mineral composition was characterized by X-ray diffraction (XRD) on a D/8 Advance XRD (Rigaku, Japan) with Cu Ka radiation between 5 and 70 degrees with a 2 deg/min scanning speed. Furthermore, the characteristics of the Al paste were measured according to the methods mentioned in JC/T 407-2008.

#### 2.3.2. Slurry Performance

The performance of the AAC slurry includes slump flow, slurry foaming and time-dependent rheological behavior. Slump flow was tested by a cone on a horizon acrylic plate after being filled with ultimate slurry, the cone was slowly lifted and the diameter of the slurry spread was measured [25].

The slurry foaming height was determined by the following process. The ultimate slurry was poured into a 250 mL measuring cylinder and transfered to an environmental control chamber at 55 ± 1 °C and 70% RH, and the volume of slurry was recorded every 2 min until it stopped expanding. Furthermore, the slurry foaming height was converted to the slurry foaming rate to demonstrate the gas expansion characteristics per volume of slurry. The conversion formula is shown as F_1_.

(1) $$F_{r}=\frac{V_{r}-V_{o}}{V_{o}}$$

F_r_: Slurry foaming rate

V_r_ (mL): Recorded slurry volume

V_o_ (mL): Original slurry volume

**Figure 2:**
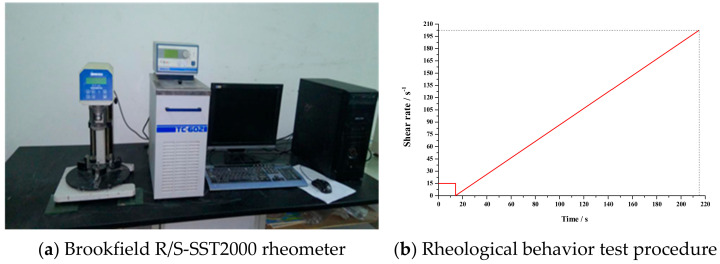
Time-dependent rheological testing device and procedure.

Time-dependent rheological behaviour was investigated on a Brookfield R/S-SST2000 rheometer (Middleboro, MA, USA) (Figure 2a) with V-72 vane spindle as a stirrer with a shear stress and viscosity range of 0.188–1.88 Pa and 104–1040 mPa, respectively. The slurry for time-dependent rheological testing was the primary slurry without the Al paste, which can avoid detection error caused by volume expansion during the testing program. Every mixture was tested at 0 min, 30 min, 60 min, 90 min and 120 min to imitate the pre-curing process. The test routine shear rate is presented in Figure 2b, rotating the spindle at 15 s^−1^ for 15 sec, then sped up evenly from 0 s^−1^ to 200 s^−1^.

#### 2.3.3. Physical-Mechanical Property

The physical-mechanical property was reflected through the measurement of bulk density and compressive strength, which were detected according to the methods stipulated in GB/T 11969-2008. Furthermore, specific strength (S) was defined to illustrate the relative strength among different density specimens, which were calculated as S = σ(MPa)/D(kg/m^3^) (σ, compressive strength; D, bulk density).

#### 2.3.4. Hydration Products

The hydration products were analyzed by mineralogical and thermal characteristics. The testing samples for these characteristics were a dry ground powder that was sifted by a 75 μm square-mesh sieve. Mineralogical characteristics of the AAC specimens were conducted on a D/8 Advance XRD with Cu Ka radiation between 5 and 70 degrees with a 2 deg/min scanning speed. The thermal characteristics of the AAC specimens’ hydration products were reflected through detection of differential scanning calorimetry (DSC) and thermal-gravimetric (TG) data on a NETZSCH STA449F3 simultaneous thermal analyzer (Selb, Germany) from ambient temperature to 1000 °C with a 5 °C/min heating rate. The detection atmosphere is composed of nitrogen and oxygen 3:1 to simulate normal air.

## 3. Results and Discussion

### 3.1. Performance and Characteristics of Quicklime and Carbide Slag AAC

#### 3.1.1. Slurry and Physical-Mechanical Performance

**Figure 3:**
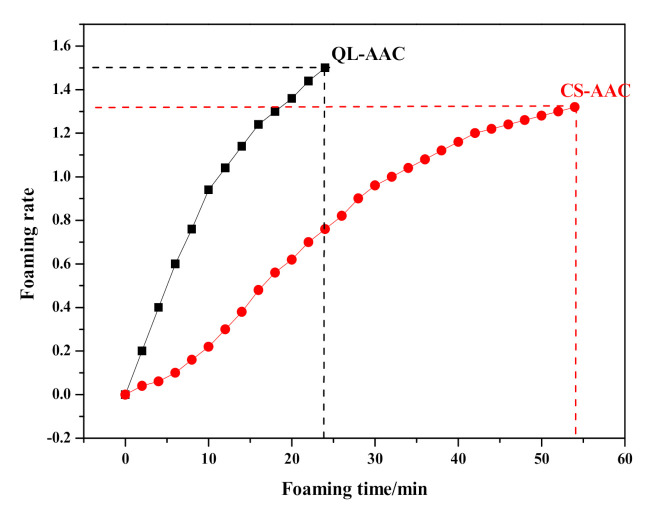
Slurry-foaming curve of mixture QL-AAC and CS-AAC.

**Figure 4:**
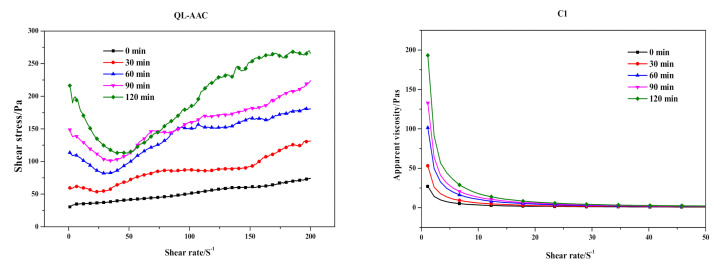

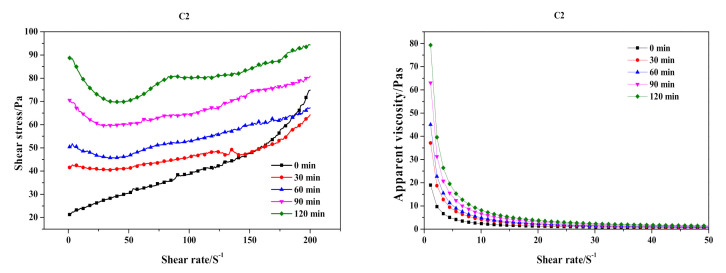
Time-dependent rheological behavior of quicklime (QL-AAC)- and carbide slag (CS-AAC)-based AAC.

Substituting carbide slag for quicklime had a serious effect on the preparation process and final products. The influence of the substitution on slurry foaming and the time-dependent rheological behavior of AAC slurry are shown in Figure 3 and Figure 4, respectively. Meanwhile, the slump flow, foaming time and physical-mechanical properties of the QL-AAC and CS-AAC mixtures are shown in Table 4. The slump flow of fresh slurry increased from 158 to 204 mm, which indicated decreasing consistency when using only carbide slag as a calcareous material. As presented in Figure 3, the slurry foaming speed obviously slowed down, and the final foaming rate decreased from 1.5 to 1.32 mL and the foaming time slowed from 24 to 54 min when quicklime was totally replaced by carbide slag, and this was attributed to the vanishing of uniform hydration heat. Furthermore, time-dependent rheological behavior revealed more characteristics of slurry thickening. At the beginning of shearing, the shear stress and apparent viscosity of QL-AAC and CS-AAC fresh slurry revealed little difference to each other. When the shear rate accelerated from 0 to 200 S^−1^, the fresh slurry of the QL-AAC mixture was close to a Bingham fluid, but the fresh slurry of CS-AAC mixture was more like a Newtonian fluid when the shear rate was less than 160 S^−1^. After exceeding 160 S^−1^, the shear stress rapidly increased. Moreover, the apparent viscosity descended to nearly 0 Pa with an increasing shear rate. Furthermore, after a period of curing, an inflection point emerged near a 30–40 S^−1^ shear rate, which was related to the disordering of a flocculent structure formed by the binding material hydration reaction [26]. The longer the curing process duration, the greater the reduction in shear stress. As the curing process proceeded, the initial value of shear stress and apparent viscosity increased at the beginning of the shearing. After curing for 120 min, the initial shear stress and apparent viscosity of quicklime-based AAC slurry reached nearly 240 Pa and 190 Pa, respectively. In contrast, the parameters of the carbide slag-based AAC slurry were only 90 Pa and 80 Pa, which indicated a much lower consistency induced by the absolute substitution of carbide slag for quicklime.

Moreover, using only carbide slag as the calcareous material also had an effect on the physical-mechanical properties of AAC products. The increasing bulk density was ascribed to decreased porosity, which caused the foaming rate to decrease. Nevertheless, the compressive strength and specific strength both decreased, which may be ascribed to the weakened generation of the two most important strength contributors to the hydration products: calcium silicate hydrate (I) (C–S–H (I)) and tobermorite. This guess was verified by further investigation and analysis on hydration products, which is found in the following discussion.

#### 3.1.2. Hydration Products

**Figure 5:**
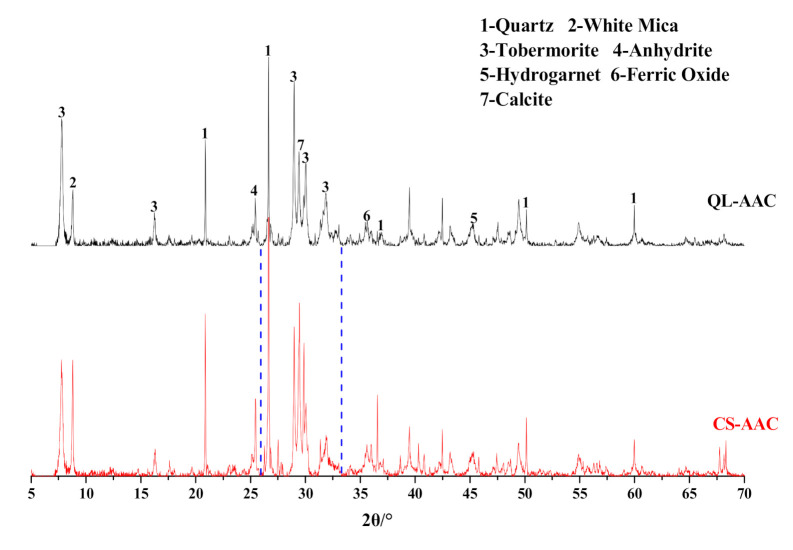
Effect of carbide slag replacing quicklime on AAC mineral composition.

The substitution of carbide slag for quicklime also influenced the hydration products of AAC specimens. The results of XRD analysis of QL-AAC and CS-AAC AAC specimens were presented in Figure 5. According to XRD analysis, the mineral species were unvaried when the calcareous material was changed. The amorphous C–S–H gel, which bonded other phases in AAC to form an interconnected network and generate strength [27,28], diffracted a broad ‘‘convex closure” between 26 and 33 deg [29]. Moreover, the intensity and area value in Table 5 revealed some remarkable regularity in the characteristic peaks of major hydration products and silicon-containing minerals in data form. The intensity and area value of the tobermorite characteristic peaks (11.3 Å, 3.08 Å) obviously diminished when carbide slag replaced quicklime, which accounts for the decreased crystallinity and content of tobermorite. Otherwise, the intensity and area value of white mica (9.93 Å) and quartz (3.34 Å) in the characteristic peak of the CS-AAC mixture was obviously much higher than that of the QL-AAC mixture, which indicated a lower consumption of white mica and quartz in carbide slag HCS-AAC than in quicklime AAC. Lower consumption of the two major silicon providers represented a lower generation of target hydration products, which was in accord with the results of the mechanical property and tobermorite characteristic peaks. This was ascribed to the pre-dissolved siliceous materials during the pre-curing stage under a large amount of hydration heat generated by quicklime. More pre-dissolving siliceous materials led to easier participation in the hydrothermal synthesis reaction in autoclave curing and more consumption of silicon containing minerals; subsequently, silicon-containing material content in the final products were reduced.

**Figure 6:**
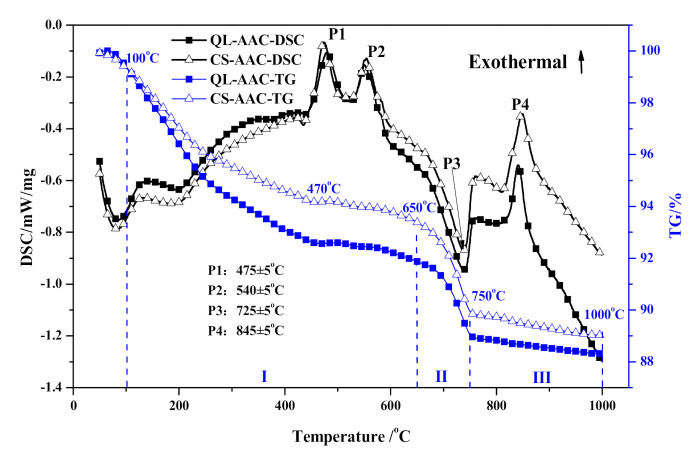
Thermal behavior analysis of QL-AAC and CS-AAC specimen.

The thermal characteristics of QL-AAC and CS-AAC specimens are presented in Figure 6 and Table 6. Taking view in the DSC curves, the endothermic effect below 100 °C was related to the evaporation of free water in the testing specimens. Moreover, 3 exothermic peaks and 1 endothermic peak were distinguishable, but no significant difference between the QL-AAC and CS-AAC mixtures could be recognized. P1 and P2 stand for the oxidizing reaction of organic matter from iron tailings according to a former study of the authors [30]. The only major endothermic peak emerged near 725 °C (P3) and corresponded to the decomposition of calcite (CaCO_3_) together with distinctive weight loss (stage II: 650–750 °C) from CO_2_ release. The last exothermic peak (P4) presented at 845 ± 5 °C, corresponded to the transformation of C–S–H (A) to β-wollastonite along with sharp shrinkage [31,32,33,34].

As the TG curves show, the thermal behavior analysis of QL-AAC and CS-AAC specimen was artificially segmented into three stages in accordance with the predominate heating reaction. Meanwhile, the weight loss of each temperature range was calculated and shown in Table 6 accordingly. The reaction from 100 to 650 °C (stage I) was caused by the dehydration of major hydration products, C–S–H (I) and tobermorite, transforming into C–S–H(A). The weight loss obviously decreased when quicklime was totally replaced by carbide slag. The weight loss variation in stage I reflected the decreased content of C–S–H(I) and tobermorite, which corresponded with the regulation of mechanical and mineralogical analysis. Stage II (650–750 °C) was mainly determined by the decomposition of calcite (CaCO_3_) in iron tailing and carbide slag, which was augmented when quicklime was substituted for carbide slag. The third stage (750–1000 °C), which related to the transformation of xonotlite and C–S–H (A) into β-wollastonite, revealed bare distinctions among different calcareous materials. Thus, the thermal characteristics analysis demonstrated analogous mineral composition and relative content with the mineralogical analysis of QL-AAC and CS-AAC specimens. Furthermore, the mechanical property decline caused by the decrease in C–S–H content and tobermorite crystallinity was proved by mineralogical and thermal characteristics analysis.

### 3.2. Coagulating Mechanism of Na_2_SO_4_ and Na_2_O·2.0SiO_2_ in Cement Paste

The main purpose of introducing a cement coagulant into HCS-AAC is to accelerate slurry thickening and make the rough-body porosity more stable by promoting the cement hydration rate. Therefore, the coagulant mechanism of Na_2_SO_4_ and Na_2_O·2.0SiO_2_ on cement must first be clarified, and then the influence mechanism on HCS-AAC, according to an analysis of the effect on HCS-AAC slurry performance, needs to be figured out, thereby providing support for coagulant application in HCS-AAC production.

The cement coagulants used in this paper are all compounds combining cationic sodium and acid ions, which have a similar behavior in cement slurry. Na_2_SO_4_ reacted with Ca(OH)_2_ generated by cement hydration to form highly dispersed calcium sulfate (CaSO_4_) and sodium hydroxide (NaOH) that were evenly distributed in the slurry (F2). CaSO_4_ and calcium aluminate (C_3_A) formed ettringite (AFt) quickly (F3) while NaOH increasing the solubility of gypsum and aluminate, thus accelerating the hydration of alite (C_3_S) and formation of AFt, which greatly sped up the hardening process. Na_2_O·2.0SiO_2_ can strongly react with Ca(OH)_2_ in a cement slurry to form calcium silicate (CaSiO_3_), NaOH and SiO_2_ gel (F4). NaOH will further promote cement clinker mineral hydration, thus making cement set and harden quickly as previously mentioned. It can be seen that Na_2_SO_4_ and Na_2_O·2.0SiO_2_ both react with Ca(OH)_2_ from cement hydration to form NaOH and further expedite the hydration of C_3_S by NaOH to realize accelerated cement coagulation [19].

(2) $${\mathrm{NaSO}}_{4}+\mathrm{Ca}{\left(\mathrm{OH}\right)}_{2}{+2H}_{2}O\to {\mathrm{CaSO}}_{4}\cdot {2H}_{2}O+2\mathrm{NaOH}$$

(3) $$3\left({\mathrm{CaSO}}_{4}\cdot {2H}_{2}O\right)+3\mathrm{CaO}\cdot {\mathrm{Al}}_{2}O_{3}\cdot {6H}_{2}{O+20H}_{2}O\to 3\mathrm{CaO}\cdot {\mathrm{Al}}_{2}O_{3}\cdot {3\mathrm{CaSO}}_{4}\cdot {32H}_{2}O$$

(4) $${\mathrm{Na}}_{2}O\cdot {\mathrm{nSiO}}_{2}+\mathrm{Ca}{\left(\mathrm{OH}\right)}_{2}\to \left(n-1\right){\mathrm{SiO}}_{2}{(\mathrm{gel})+\mathrm{CaSiO}}_{3}+2\mathrm{NaOH}$$

In the HCS-AAC slurry, Ca(OH)_2_ in carbide slag and CaSO_4_ in PG will dissolve, resulting in a higher initial concentration of hydroxyl and sulfate ions, which is the main difference with a cement slurry. Therefore, analyzing the influence of Ca(OH)_2_ and CaSO_4_ on the coagulation-promoting effect of Na_2_SO_4_ and Na_2_O·2.0SiO_2_ in HCS-AAC slurry is a key issue to be discussed in subsequent experiments.

### 3.3. Slurry Performance Regulating Effect of Cement Accelerator on HCS-AAC

#### 3.3.1. Regulating Effect of Na_2_SO_4_

**Figure 7:**
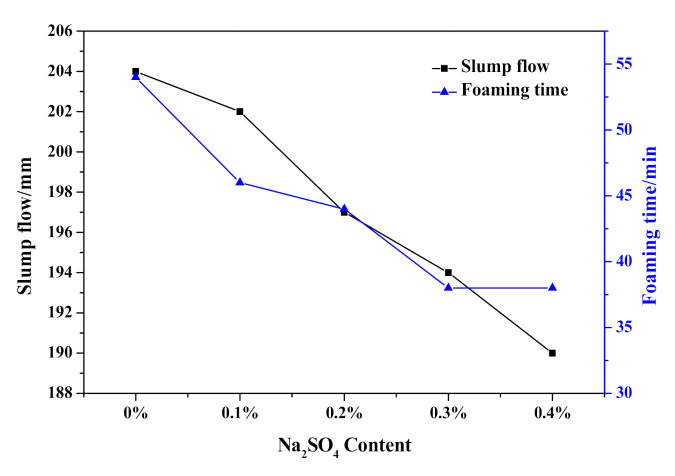
Effect of Na_2_SO_4_ content on slump flow and foaming time of HCS-AAC slurry.

**Figure 8:**
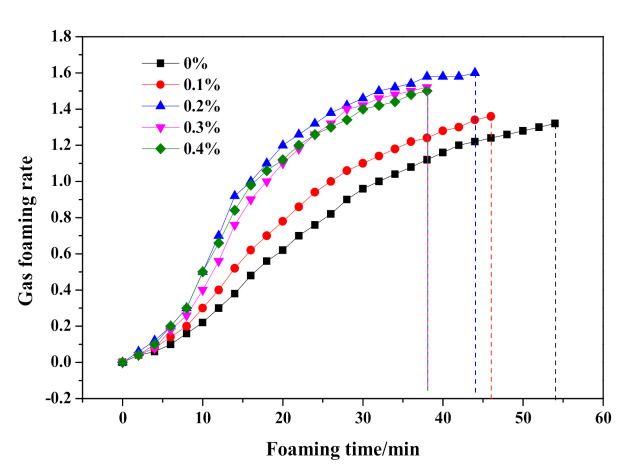
Effect of Na_2_SO_4_ content on slurry foaming property of HCS-AAC slurry.

As shown in Figure 7, the slump flow continuously fell from 204 to 190 mm, which indicated an increasing initial consistency of HCS-AAC slurry with the addition of Na_2_SO_4_. Meanwhile, the termination time of the slurry foaming process also shortened from 53 to 34 min. With further analysis of the foaming process in Figure 8, the slurry foaming speed significantly accelerated after 5 min and gradually increased with the addition of Na_2_SO_4_. The foaming volume reached its maximum with the addition of 0.2% Na_2_SO_4_ and decreased slightly after the Na_2_SO_4_ content was increased. It can be seen from the slurry foaming curves that the increase of sodium sulfate had an obvious effect on the acceleration of the slurry foaming rate and the shortening of the slurry foaming time, so the slurry foaming curve is close to the range recommended by Jineng Zhang [5].

**Figure 9:**
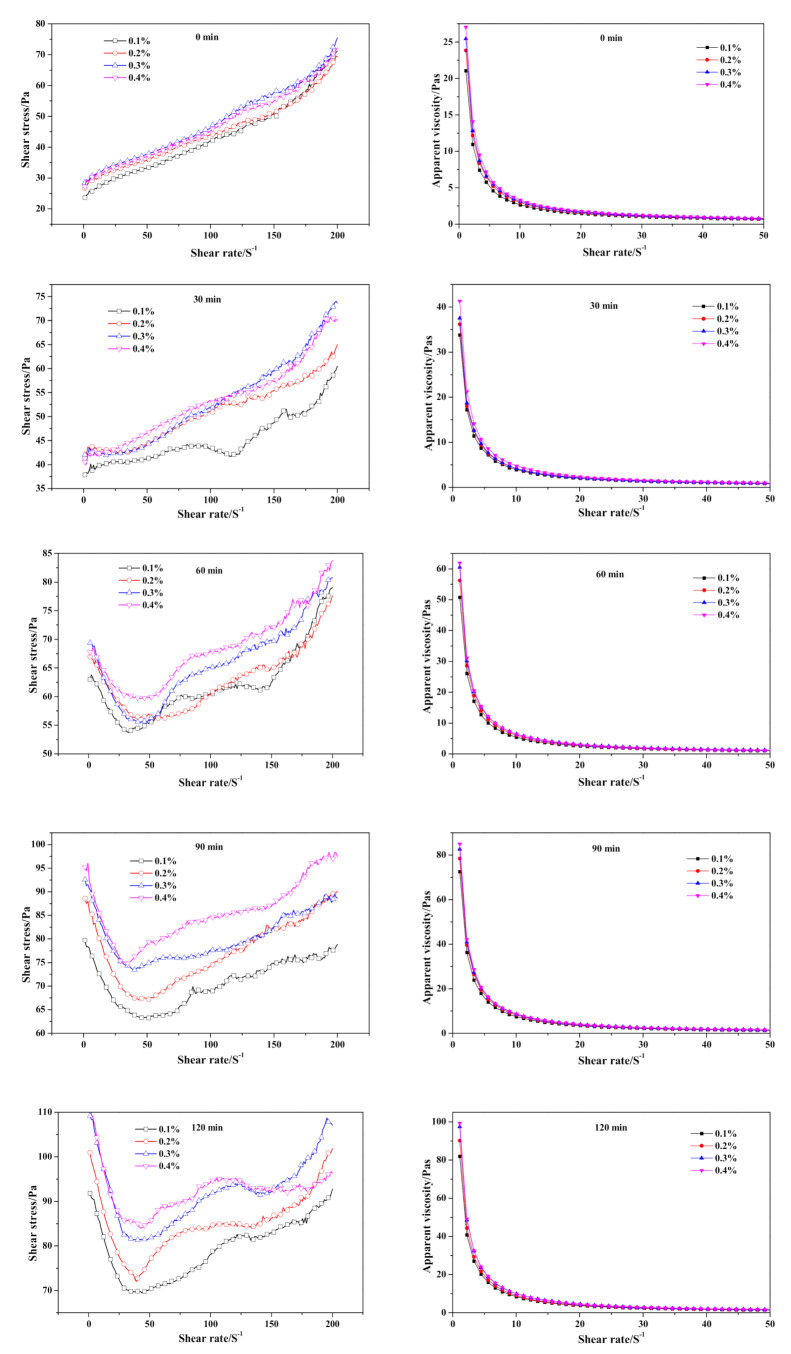
Effect of Na_2_SO_4_ content on neat slurry time-dependent rheological behavior.

As time-dependent shear stress curves show in Figure 9, the initial shear stress of fresh slurry (0 min) was barely influenced by the dose of Na_2_SO_4_. As the pre-curing went on, the disparity of shear stress among slurries of differing Na_2_SO_4_ content gradually widened and increased with the addition of Na_2_SO_4_ content. The inflection point caused by the destruction of the flocculent structure appeared in the 60 min curves, which was much deeper than shear stress curve of the CS-AAC slurry (Figure 4) at 60 min, indicating the thickening effect of the Na_2_SO_4_ addition. Otherwise, the initial apparent viscosity value also revealed a similar law with shear stress, as it increased successively with the addition of Na_2_SO_4_.

#### 3.3.2. Regulating Effect of Na_2_O·2.0SiO_2_

**Figure 10:**
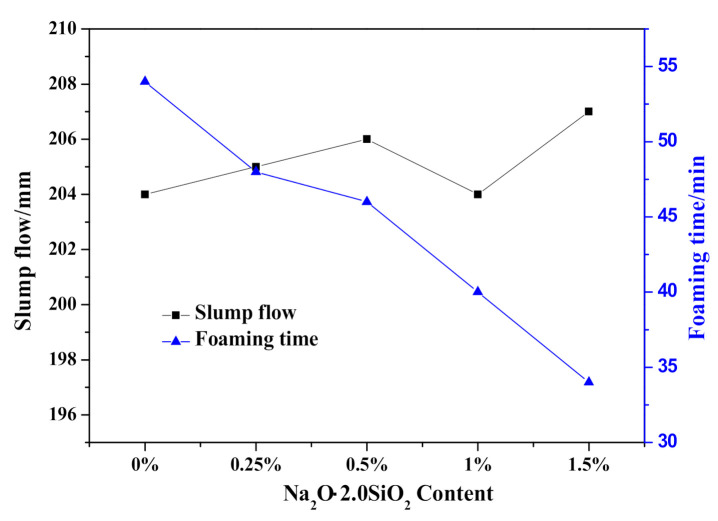
Effect of Na_2_O·2.0SiO_2_ content on slump flow and foaming time of HCS-AAC slurry.

**Figure 11:**
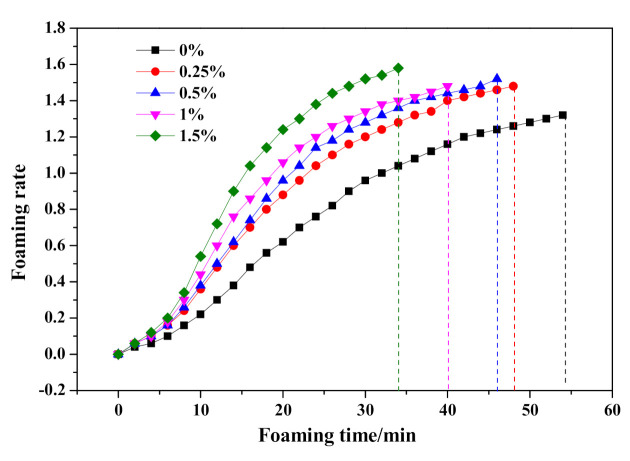
Effect of Na_2_O·2.0SiO_2_ content on slurry foaming property of HCS-AAC slurry.

The effect of Na_2_O·2.0SiO_2_ content on the HCS-AAC slurry slump flow and slurry foaming property are shown in Figure 10 and Figure 11, respectively. As presented in Figure 10, the slump flow constant is a small range (204–208 mm), meanwhile, the slurry foaming termination time successively shortened with increasing Na_2_O·2.0SiO_2_ content in sequence, which can also be seen in the foaming curves in Figure 11. In view of the slurry foaming process with the incremental dosage of Na_2_O·2.0SiO_2_, the expansion speed of the rough body increased and the ultimate foaming rate increased gradually, which indicated that the addition of Na_2_O·2.0SiO_2_ accelerated the gassing of Al powder, and its accelerating effect on the foaming process was stronger than that on the thickening process which resulted in the increase of the ultimate foaming rate.

**Figure 12:**
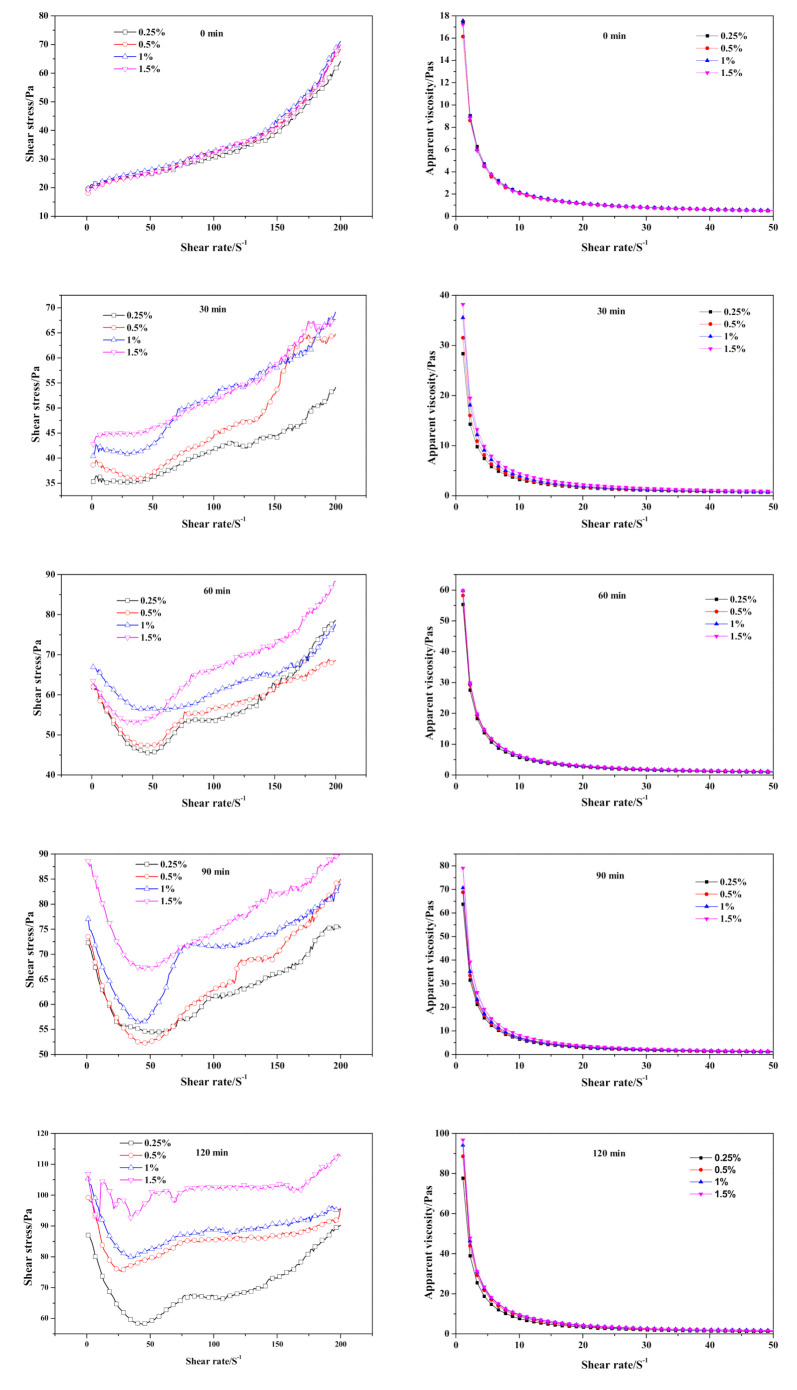
Effect of Na_2_O·2.0SiO_2_ content on neat slurry time-dependent rheological behavior.

Regarding the time-dependent rheological behavior exhibited in Figure 12, it was hard to tell the difference in shear stress and apparent viscosity of fresh slurry (0 min) as Na_2_O·2.0SiO_2_ content varied. As curing proceeded, the initial shear stress and apparent viscosity value increased; meanwhile, the disparity induced by the addition of Na_2_O·2.0SiO_2_ gradually widened synchronously. The inflection point exhibited at the 30 min curves, was earlier than the CS-AAC mixture slurry in Figure 6, indicating that the flocculation structure for increasing slurry consistency increased with the addition of Na_2_O·2.0SiO_2_, which had a similar accelerating effect as Na_2_SO_4_ had on the slurry foaming and curing process.

#### 3.3.3. Effect Mechanism Analysis

The accelerating effect of Na_2_SO_4_ on the slurry foaming process is due to the fact that the Al(OH)_3_ produced by Al paste reacts with water in slurry to form a gelatinous substance (F5) that adheres to the surface of the Al ion after formation and prevents the new surface of Al from further reacting with water. In a conventional AAC slurry, the gelatinous Al(OH)_3_ can react with Ca(OH)_2_ to form Ca(AlO_2_)_2_ (F6), so that the Al ion can keep on reacting with water. With addition of Na_2_SO_4_, one of the products NaOH can promote is the decomposition reaction of Al(OH)_3_ (F7), thus accelerating the gassing reaction of Al particles. The accelerating mechanism of Na_2_O·2.0SiO_2_ on HCS-AAC slurry foaming was similar to Na_2_SO_4_.

(5) $${2\mathrm{Al}+6H}_{2}O\to 2\mathrm{Al}{\left(\mathrm{OH}\right)}_{3}{+3H}_{2}↑$$

(6) $$2\mathrm{Al}{\left(\mathrm{OH}\right)}_{3}{+\mathrm{Ca}(\mathrm{OH})}_{2}\to {\mathrm{Ca}(\mathrm{AlO}}_{2}{)}_{2}{+4H}_{2}O$$

(7) $$\mathrm{Al}{\left(\mathrm{OH}\right)}_{3}+\mathrm{NaOH}\to {\mathrm{NaAlO}}_{2}{+2H}_{2}O$$

From the perspective of the coagulating effect, both Na_2_SO_4_ and Na_2_O·2.0SiO_2_ have a slight coagulation-promoting effect on an HCS-AAC slurry. After adding 0.4% Na_2_SO_4_ and 1.5% Na_2_O·2.0SiO_2_, the apparent viscosity of the slurry increased from 80Pa to nearly 100Pa in 120 min. However, according to the comparison with the apparent viscosity development curves in Figure 5 (QL-AAC), the coagulation effect was far from the target value of a traditional quicklime AAC slurry. This was ascribed to the low cement content (only 10%) in the HCS-AAC slurry and the slurry’s uniform dispersion state. Although hydration was accelerated, the formation of a mesoscopic hydration-product frame structure was not significantly accelerated, which was manifested in the slow consistency growth at the macroscopic level. It is speculated that other coagulants that can accelerate cement hydration may have similar effects on the setting rate of an HCS-AAC slurry.

Therefore, it can be seen that Na_2_SO_4_ and Na_2_O·2.0SiO_2_ can effectively accelerate the slurry-foaming process, but the promotional effect on slurry thickening is inconspicuous. It is suggested that, based on the previous research results of the authors on AAC slurry performance, combining binding material modification, cement coagulant and rapid hardening cement in HCS-AAC could realize the common regulation of slurry foaming and thickening process [12].

### 3.4. Effect of Cement Accelerator on HCS-AAC Physical-Mechanical Properties

#### 3.4.1. Effect of Na_2_SO_4_

**Figure 13:**
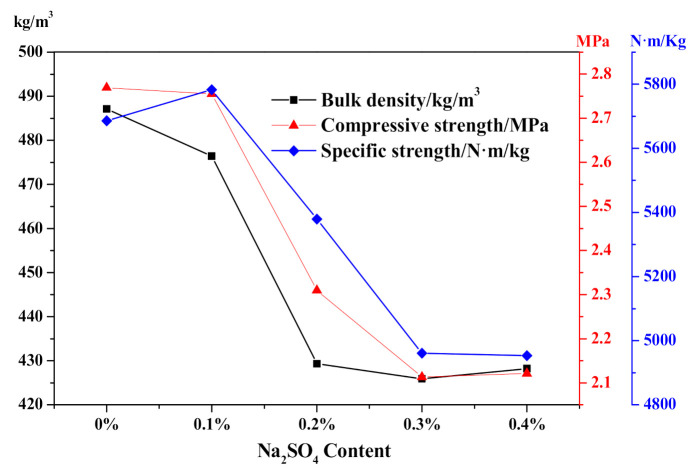
Effect of Na_2_SO_4_ content on HCS-AAC physical-mechanical property.

Accoding to the effect of Na_2_SO_4_ content on HCS-AAC physical-mechanical properties as presented in Figure 13, bulk density decreased from approximately 480 to 425 kg/m^3^, which was in agreement with the influence on the slurry foaming process. Meanwhile, the compressive strength decreased from 2.77 to 2.1 MPa, and the specific strength also shown a downward trend, corresponding to the decline of bulk density.

#### 3.4.2. Effect of Na_2_O·2.0SiO_2_

**Figure 14:**
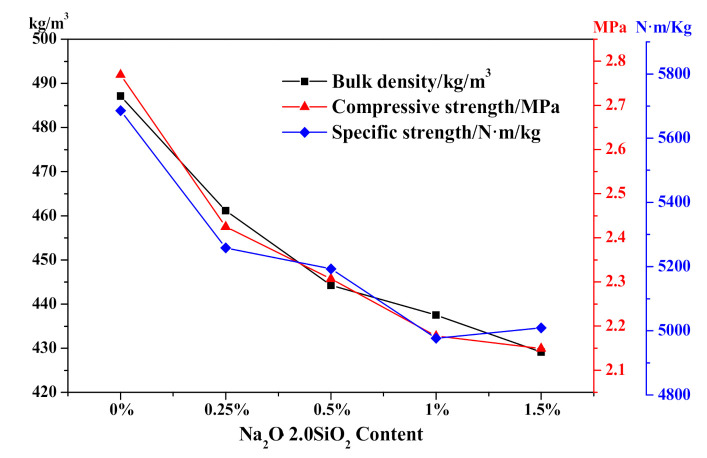
Effect of Na_2_O·2.0SiO_2_ content on HCS-AAC physical-mechanical property.

Regarding the physical-mechanical properties of different Na_2_O·2.0SiO_2_ content HCS-AAC specimens as shown in Figure 14, as the Na_2_O·2.0SiO_2_ content increased from 0% to 1.5%, the bulk density successively decreased from 490 to 430 kg/m^3^ which was consistent with the increment of the ultimate foaming rate. Meanwhile, the compressive strength of HCS-AAC samples rapidly fell from 2.77 to 2.42 MPa when a dose of 0.25% Na_2_O·2.0SiO_2_ was added. With more addition, the compressive strength and specific strength decreased synchronously with bulk density, which almost reached the B04, A2.0 (bulk density ≤ 425 kg/m^3^, a compressive strength ≥ 2.0 MPa)-grade product stipulated in GB 11968-2006. The effect of Na_2_O·2.0SiO_2_ on the physical-mechanical properties of HCS-AAC is similar to that of Na_2_SO_4_.

The mechanical properties varied with the fluctuation of bulk density; in other words, the bulk density and compressive strength of HCS-AAC are mainly determined by the slurry foaming process. Furthermore, the effect of a cement coagulant on hydration products will be analyzed in detail in the next section.

### 3.5. Effect of Cement Accelerator on HCS-AAC Hydration Products

#### 3.5.1. Effect of Na_2_SO_4_

**Figure 15:**
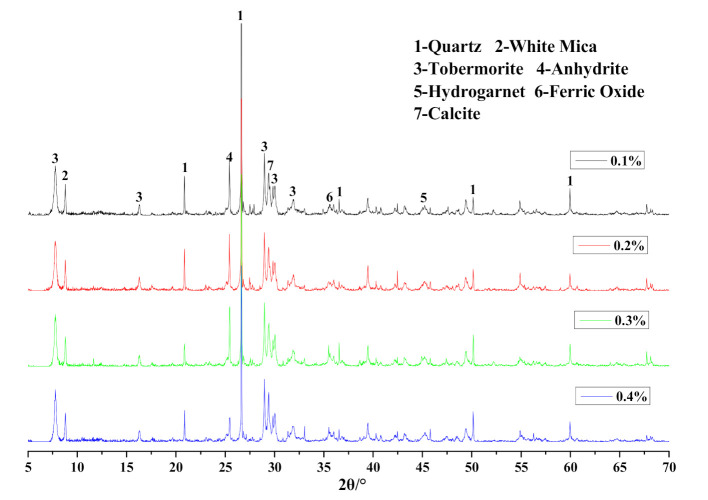
XRD patterns of HCS-AAC samples with different Na_2_SO_4_.

The mineralogical analysis results of different Na_2_SO_4_ content in HCS-AAC specimens is shown in Figure 15 and Table 7. The mineral composition was unchanged; meanwhile, the parameters of tobermorite varied only slightly with the increase in Na_2_SO_4_ content, which indicated that the variation of Na_2_SO_4_ content under 0.4% had scarcely any influence on the generation of tobermorite.

**Figure 16:**
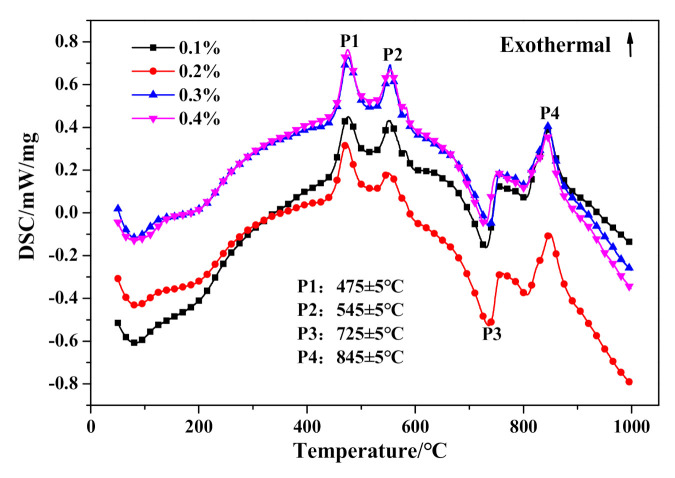
DSC curves of HCS-AAC with different Na_2_SO_4_ content.

**Figure 17:**
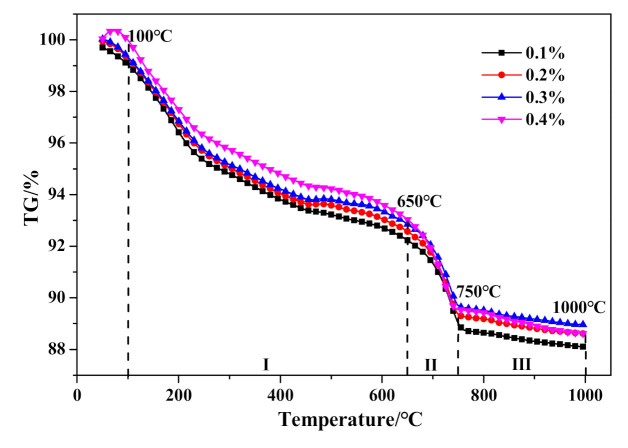
TG curves of HCS-AAC with different Na_2_SO_4_.

With further investigation of thermal characteristics, the characteristic peaks of DSC curves in Figure 16 showed barely any distinction between HCS-AAC specimens with varied Na_2_SO_4_ content. Similarly, the TG course (Figure 17) and weight changes of each characteristic stage (Table 8) showed hardly any discernible difference or regularity. To sum up, the addition of Na_2_SO_4_ hardly influences the generation of C–S–H (I) and the crystallinity of tobermorite.

#### 3.5.2. Effect of Na_2_O·2.0SiO_2_

**Figure 18:**
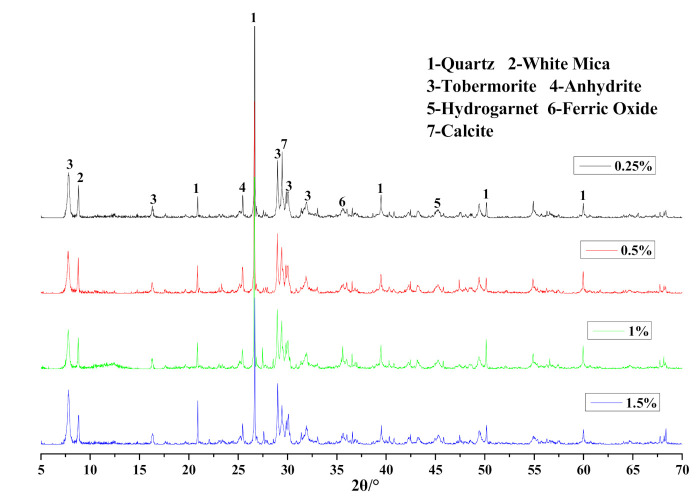
XRD patterns of HCS-AAC samples with different Na_2_O·2.0SiO_2_.

As mineralogical analysis demonstrated in Figure 18, the position of diffraction peaks did not shift with the employment of Na_2_O·2.0SiO_2_. However, the intensity and area value of the corresponding tobermorite characteristic peaks in Table 9 gradually rose with increasing Na_2_O·2.0SiO_2_ content, which indicated the accelerating effect on the generation and crystalization of tobermorite.

**Figure 19:**
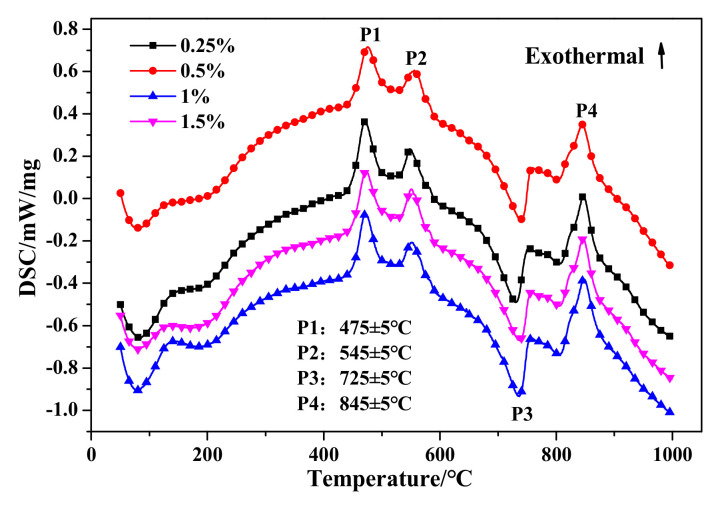
DSC curves of HCS-AAC with different amounts of Na_2_O·2.0SiO_2_.

**Figure 20:**
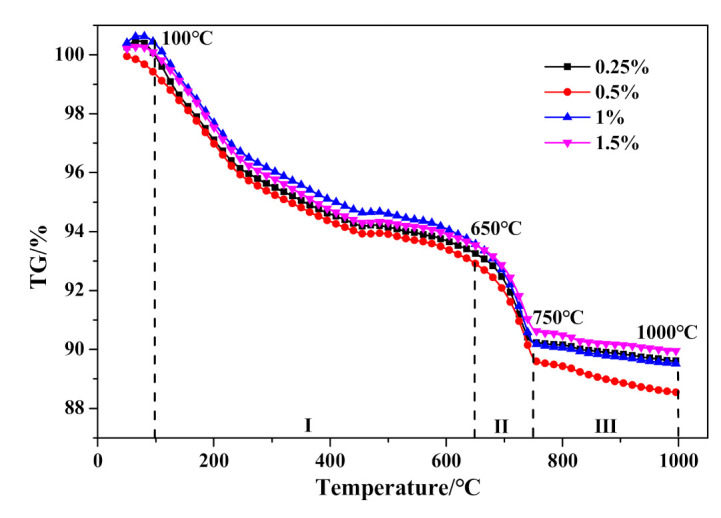
TG curves of HCS-AAC with different amounts of Na_2_O·2.0SiO_2_.

The DSC and TG results are presented in Figure 19 and Figure 20, respectively. Firstly, no significant variation can be drawn from the DSC curves exhibited in Figure 19. Otherwise, as presented in Table 10, the weight loss in stage I would gradually increase with the increase of Na_2_O·2.0SiO_2_ content, which indicated that Na_2_O·2.0SiO_2_ promoted the generation and crystalization of C–S–H (I), ultimately resulting in the strengthening of a pore-segmented rampart. Therefore, although the bulk density of HCS-AAC products decreased rapidly, the decline in compressive strength was relatively slow. The weight change between stage II and stage III showed no inevitable regularity with increasing Na_2_O·2.0SiO_2_ content.

#### 3.5.3. Effect Mechanism Analysis

According to the above analysis of hydration products, the addition of Na_2_SO_4_ hardly influenced the generation of C–S–H (I) or the crystallinity of tobermorite, while Na_2_O·2.0SiO_2_ promoted the generation of strength-contributing minerals. The influence mechanism can be traced back to the production of a cement coagulant in an alkaline slurry in Section 3.2. As equations F2 and F4 showed, except for the common product NaOH, Na_2_SO_4_ produced CaSO_4_·2H_2_O in the slurry while the products of Na_2_O·2.0SiO_2_ and Ca(OH)_2_ include gelatinous SiO_2_ and CaSiO_3_. Since the 3% PG added to the HCS-AAC provided sufficient CaSO_4_·2H_2_O for a hydrothermal synthesis reaction, a small amount of calcium sulfate generated by Na_2_SO_4_ had little influence on generating the main strength-contributing hydration products. Nevertheless, one of the products between Na_2_O·2.0SiO_2_ and Ca(OH)_2_ is the high-activity gelatinous SiO_2_, which can rapidly combine with Ca(OH)_2_ to form C–S–H gel and further promote the formation of C-S-H(I) and tobermorite with increased temperature, thus improving the strength of the pore-segmented rampart.

## 4. Conclusions

This article addressed the problem of HCS-AAC pre-curing caused by the absolute substitution of carbide slag for quicklime, by introducing the commonly used cement accelerators Na_2_SO_4_ and Na_2_O·2.0SiO_2_ to adjust slurry foaming and thickening. Measuring the feasibility in HCS-AAC by a comprehensive impact analysis of slurry performance, physical-mechanical properties and hydration products. Synthesizing all of the above research, the following conclusions were reached:

(1) Na_2_SO_4_ and Na_2_O·2.0SiO_2_ can effectively accelerate the slurry foaming process, but the promotion of the slurry thickening process is inconspicuous. Thus, the formation stability of a porous structure rough body is adversely affected. It is recommended that a cement coagulant and rapid hardening cement be used together in an HCS-AAC to realize the common regulation of slurry foaming and thickening.

(2) With the increase in cement coagulant content, the compressive strength of an HCS-AAC obviously fell, which corresponded to the steady fall of bulk density and is mainly ascribed to the acceleration of slurry foaming.

(3) The dosing of Na_2_SO_4_ under 0.4% had little effect on the generation of strength-contributing hydration products. Nevertheless, the addition of Na_2_O·2.0SiO_2_ in a carbide slag HCS-AAC had a superior accelerating effect on C–S–H generation and crystalization than Na_2_SO_4_, which contributed to the high-activity gelatinous SiO_2_ generated by the reaction between Na_2_O·2.0SiO_2_ and Ca(OH)_2_.

## Figures and Tables

**Table 1:** Chemical components of raw materials (%).

Raw Materials	SiO_2_	Al_2_O_3_	CaO	Fe_2_O_3_	MgO	Na_2_O	K_2_O	SO_3_	CO_2_	LOI
PO 42.5	17.76	3.94	61.11	4.04	1.78	—	0.29	3.52	6.32	0.73
Carbide slag	2.57	1.88	65.03	0.09	0.14	0.09	—	0.67	—	28.31
Quicklime	2.78	1.02	73.64	0.73	1.45	—	0.13	0.33	12.7	6.94
Iron tailing	42.90	10.75	12.97	7.51	7.10	2.06	1.96	9.04	—	4.48
Quartz tailing	93.23	1.68	0.33	0.56	0.14	—	0.64	—	—	0.78
PG	10.64	1.22	25.39	0.54	0.19	0.23	0.50	36.56	—	22.91

**Table 2:** Granularity characteristics of raw materials.

Raw Materials	PO 42.5	Quicklime	Carbide Slag ^a^	Siliceous Mix Powder ^b^
Specific surface area (m^2^/kg)	336	328	284	306
80 μm screen residue weight (%)	10.8	9.3	20.03	14.9

^a^ Carbide slag was ground for 20 min in a φ500 mm × 500 mm cylinder ball mill. ^b^ Siliceous mix powder was 20 min ball mill ground mixture of iron tailing, quartz tailing and PG dry powder according to the mass ratio 28:28:3.

**Table 3:** Mixture of control groups.

NO	PO 42.5	Calcareous Material		Siliceous Material		PG	Al Paste	W/P
		Quicklime	Carbide Slag	Iron Tailing	Quartz Tailing			
^a^ QL-AAC	10%	31%	—	28%	28%	3%	0.14%	0.54
^b^ CS-AAC	10%	—	31%	28%	28%	3%	0.14%	0.54

^a^ QL-AAC: Comparison group; ^b^ CS-AAC: Control group.

**Table 4:** Slurry performance and physical-mechanical properties of quicklime and carbide slag AAC.

Properties	Slump Flow/mm	Foaming Time/min	Bulk Density/kg/m^3^	Compressive Strength/MPa	Specific Strength /N·m/kg
QL-AAC	158	24	497.46	2.94	5910
CS-AAC	204	54	514.41	2.77	5385

**Table 5:** Parameters of tobermorite, white mica and quartz characteristic peaks of QL-AAC and CS-AAC specimen.

Mineral-d(A)	Tobermorite 11.3 Å		Tobermorite 3.08 Å		White Mica-9.93 Å		Quartz-3.34 Å	
Group	QL-AAC	CS-AAC	C1QL-AAC	CS-AAC	QL-AAC	CS-AAC	QL-AAC	CS-AAC
Intensity/cps	2450	2115	3140	2618	1011	2198	3618	11,299
Area	37,935	31,301	34,622	28,139	10,427	14,292	20,304	45,458

**Table 6:** Weight loss ratio of the temperature ranges in Figure 6.

Temperature Range/°C	Weight Loss/%	
	QL-AAC	CS-AAC
I-(100–650)	−7.53103	−5.95798
II-(650–750)	−2.82582	−3.43866
III-(750–1000)	−0.73270	−0.92116

**Table 7:** Tobermorite characteristic peaks parameter of HCS-AAC with different Na_2_SO_4_.

d(A)	11.3 Å				3.08 Å			
Content/%	0.1	0.2	0.3	0.4	0.1	0.2	0.3	0.4
Intensity/cps	2547	2708	2711	2566	2994	3275	3242	3098
Area	37,464	37,947	38,420	35,861	29,845	32,383	33,084	30,835

**Table 8:** Weight-loss ratio of the temperature ranges in Figure 17.

Temperature Range/°C	Weight Loss/%			
	0.1%	0.2%	0.3%	0.4%
I-(100–650)	−6.46143	−6.47767	−6.47988	−6.48829
II-(650–750)	−3.34939	−3.20937	−3.15367	−3.05467
III-(750–1000)	−0.7884	−0.75968	−0.74335	−0.74236

**Table 9:** Tobermorite characteristic peak parameters of HCS-AAC with different amounts of Na_2_O·2.0SiO_2_.

d(A)	11.3 Å				3.08 Å			
Content/%	0.25	0.5	1	1.5	0.25	0.5	1	1.5
Intensity/cps	2442	2598	2663	2830	2972	3027	3064	3165
Area	36,126	36,649	36,835	43,120	30,087	30,138	30,656	31,192

**Table 10:** Weight loss ratio of the temperature ranges in Figure 20.

Temperature Range/°C	Weight Loss/%			
	0.25%	0.5%	1%	1.5%
I-(100–650)	−6.41159	−6.47184	−6.67727	−6.84848
II-(650–750)	−3.01298	−3.21536	−3.35119	−2.84811
III-(750–1000)	−0.63945	−1.1606	−0.70715	−0.74665

## Data Availability

Data are contained within the article or supplementary material.
